# Enhanced thermophysical properties via PAO superstructure

**DOI:** 10.1186/s11671-016-1802-1

**Published:** 2017-01-11

**Authors:** Zahra Pournorouz, Amirhossein Mostafavi, Aditya Pinto, Apparao Bokka, Junha Jeon, Donghyun Shin

**Affiliations:** 1 Mechanical and Aerospace Engineering, The University of Texas at Arlington, Arlington, TX 76019-0023 USA; 2 Chemistry and Biochemistry, The University of Texas at Arlington, Arlington, TX 76019-0065 USA

**Keywords:** PAO (polyalphaolefin), Heat capacity, Nanofluid, Ethylen glycol, Thermal conductivity, Viscosity

## Abstract

For the last few years, molten salt nanomaterials have attracted many scientists for their enhanced specific heat by doping a minute concentration of nanoparticles (up to 1% by weight). Likewise, enhancing the specific heat of liquid media is important in many aspects of engineering such as engine oil, coolant, and lubricant. However, such enhancement in specific heat was only observed for molten salts, yet other engineering fluids such as water, ethylene glycol, and oil have shown a decrease of specific heat with doped nanoparticles. Recent studies have shown that the observed specific heat enhancement resulted from unique nanostructures that were formed by molten salt molecules when interacting with nanoparticles. Thus, such enhancement in specific heat is only possible for molten salts because other fluids may not naturally form such nanostructures. In this study, we hypothesized such nanostructures can be mimicked through in situ formation of fabricated nano-additives, which are putative nanoparticles coated with useful organic materials (e.g., polar-group-ended organic molecules) leading to superstructures, and thus can be directly used for other engineering fluids. We first applied this approach to polyalphaolefin (PAO). A differential scanning calorimeter (DSC), a rheometer, and a customized setup were employed to characterize the heat capacity, viscosity, and thermal conductivity of PAO and PAO with fabricated nano-additives. Results showed 44.5% enhanced heat capacity and 19.8 and 22.98% enhancement for thermal conductivity and viscosity, respectively, by an addition of only 2% of fabricated nanostructures in comparison with pure PAO. Moreover, a partial melting of the polar-group-ended organic molecules was observed in the first thermal cycle and the peak disappeared in the following cycles. This indicates that the in situ formation of fabricated nano-additives spontaneously occurs in the thermal cycle to form nanostructures. Figure of merit analyses have been performed for the PAO superstructure to evaluate its performance for heat storage and transfer media.

## Background

**Fig. 1:**
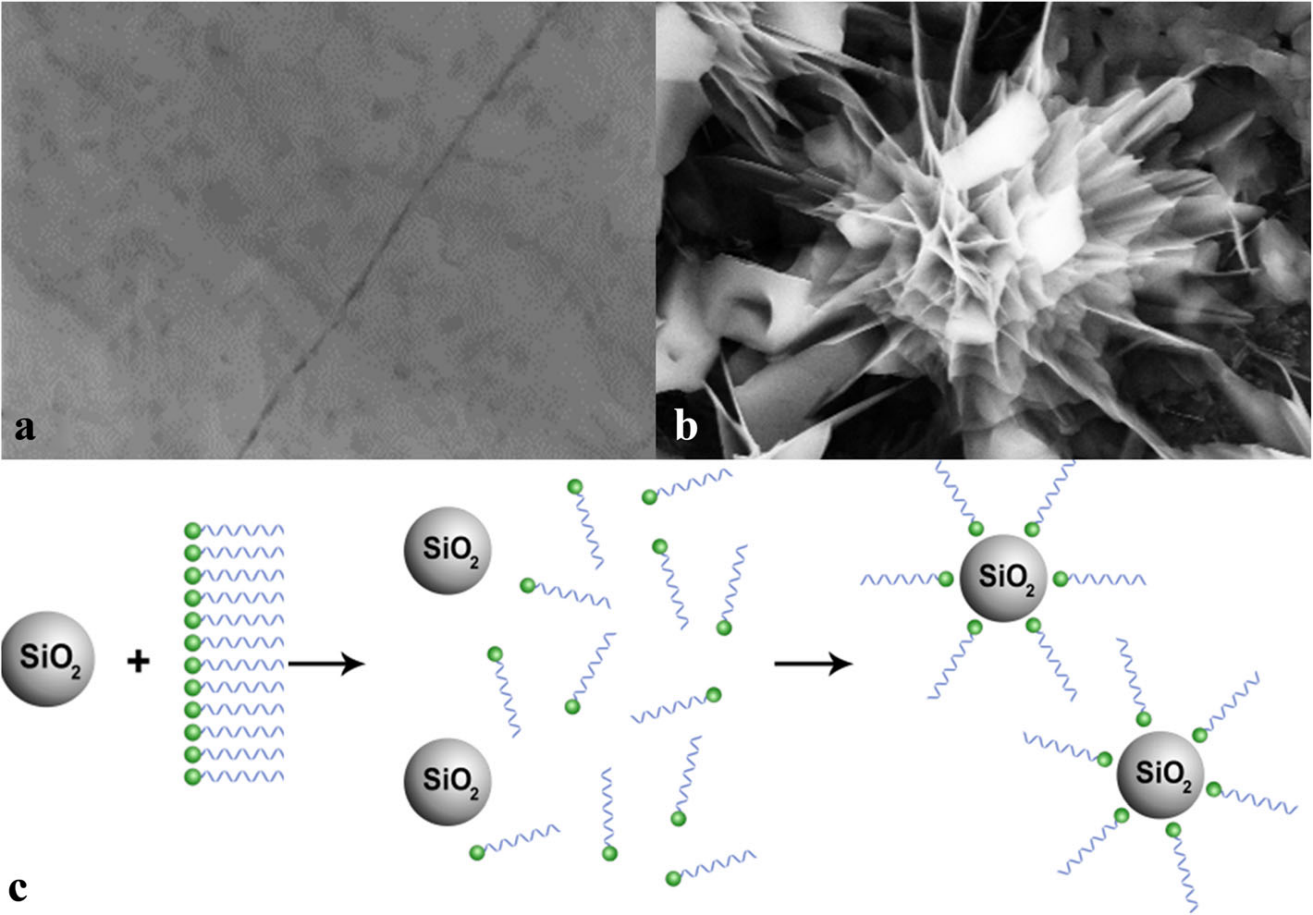
**a** A pure molten salt before dispersing nanoparticles in the previous studies. **b** A nanostructure formed in the molten salt (**a**) after dispersing nanoparticles [19, 33, 34]. The nanostructure formation was only reported for the molten salt. **c** A schematic of the proposed in situ formation of nanostructure by fabricated nano-additives

Nanoparticle suspensions in a liquid (termed as “nanofluid”) were first reported by Choi [1] in 1995 and studied for decades by many scientists and engineers for their enhancement in effective thermal conductivity [1–7]. Several mechanisms have been proposed to explain such enhanced thermal conductivity, including Brownian motion of nanoparticles, liquid molecule layering around nanoparticles, heat transfer within nanoparticles, and percolation network by aggregated nanoparticles [8–14]. While a number of studies have been reported for the enhanced effective thermal conductivity of nanofluids, controversial results have been reported for specific heat of nanofluids. Conventional water- and organic solvent-based nanofluids have shown a decrease of specific heat with the addition of nanoparticles [15–17]. For instance, silica nanoparticle dispersions in water decreased the specific heat of water by 12% with 10 vol.% nanoparticle concentration [15]. The specific heat of water decreased by 40% with 21.7 vol.% addition of alumina nanoparticles [16]. A mixture of water and ethylene glycol showed a 20% decrease of specific heat with 7 vol.% addition of ZnO nanoparticles [17]. These results showed a good agreement with the conventional effective specific heat model as follows [18]:

1 $$ {\mathrm{c}}_{\mathrm{p},\mathrm{n}\mathrm{f}}=\frac{m_{\mathrm{np}}{\mathrm{c}}_{\mathrm{p},\mathrm{n}\mathrm{p}}+{m}_f{\mathrm{c}}_{\mathrm{p},\mathrm{b}\mathrm{f}}}{m_{\mathrm{np}}+{m}_{\mathrm{bf}}} $$

where c_p_ is specific heat and *m* is mass. Subscripts nf, np, and bf denote nanofluid, nanoparticle, and base fluid, respectively. While water-based nanofluids consistently showed a decrease of specific heat with the addition of nanoparticles, molten salt eutectic showed an increase of specific heat with the addition of nanoparticles [19–32]. Since the first demonstration of enhanced specific heat of molten salt eutectic with doped nanoparticles in 2011 [19, 20], a number of studies have shown similar enhancement of specific heat for various molten salt mixtures (BaCl_2_-CaCl_2_-LiCl-NaCl, NaNO_3_-KNO_3_, LiNO_3_-NaNO_3_-KNO_3_, Li_2_CO_3_-K_2_CO_3_, etc.) [21–26]. The nanoparticles embedded for the molten salts typically have lower specific heat than those of the molten salts, and the conventional density-weighted model (Eq. 1) could not predict the enhanced specific heat. This implies that a distinct heat storage mechanism may exist other than nanoparticles and molten salts which is in turn responsible for the enhanced specific heat. According to recent studies [33, 34], molten salt molecules are likely to form a fractal-like nanostructure when a nanoparticle is dispersed. It is, however, still unclear how these fractal-like nanostructures are formed. Thus far, it was proposed that this phenomenon might be due to a unique feature of salt eutectic (i.e., a mixture of two or more to lower its melting point) and electrostatic interaction of salt molecule to a nanoparticle (which differently interacts with each type of salt) [33, 34]. Due to the difference in electrostatic force between each salt to a nanoparticle, separated salt molecules crystallize on the nanoparticle surface which functions as a nucleation point and grow to form a fractal-like nanostructure [33, 34]. Other possible mechanisms may include polarization of molten salt ions [35], multicomponent diffusional mechanisms of molten salts [36], or a combination effect of both. These fractal-like nanostructures have enhanced specific heat due to their extremely large interfacial surface area to their volume. Several studies have analytically and experimentally shown the effect of an extremely enlarged specific surface area on their effective specific heat [37–39]. Qiao et al. [32] successfully performed molecular dynamics simulations to predict such enhanced specific heat of LiNO_3_-NaNO_3_-KNO_3_ when doped with SiO_2_ nanoparticles. However, such fractal-like nanostructures can only be formed by molten salt molecules and may not naturally form in other non-salt fluids. This may explain why water, oil, and ethylene glycol-based nanofluids have shown a decrease of specific heat with nanoparticles [15–17]. Hence, it is necessary to explore the applicability of the fractal-like nanostructure into more common engineering fluids. In this study, we hypothesized that such nanostructures can be mimicked through the in situ formation of fabricated nano-additives, produced by nanoparticles which are coated with superstructure (e.g., polar-group-ended organic molecules) and thus can be simply doped into other engineering fluids. We first applied this approach to polyalphaolefin (PAO) and silica nanoparticles, and hydroxy-ended poly (ethylene glycol) (MW ca. 1400) (termed as “PBP”) was used to form an artificial nanostructure on a nanoparticle. Specifically, we envisioned that doping with the fabricated nano-additives and the nanoparticles together into PAO can initiate in situ formation of nanostructure, thereby enhancing the specific heat of PAO together with other properties (i.e., thermal conductivity and viscosity). Subsequently, a figure of merit analysis has been performed for the PAO nanostructure to evaluate its performance for heat storage and transfer media. A simple schematic of the proposed synthesis is illustrated in Fig. 1.

## Methods

Silica nanoparticles and hydroxy-ended poly (polyethylene-block-poly) (MW ca. 1400) (termed as “PBP”) were used to form an artificial nanostructure on a nanoparticle. Specifically, we envisioned that doping with the fabricated nano-additives (i.e., PBP) and the nanoparticles together into PAO can initiate in situ formation of superstructure, which acts like the fractal-like nanostructure of molten salt nanofluids, thereby enhancing the specific heat of PAO. A simple schematic of the proposed synthesis has been illustrated in Fig. 1. All three key materials (PAO-68, polyethylene-block-poly, and silica nanoparticles (10 nm; amorphous)) were procured by Behr Hella Service (Schwäbisch, Germany), Sigma-Aldrich, and Meliorum Technology, Inc., respectively.

The general procedure for preparing the sample is as follows: 98% PAO-68 (Behr Hella Service GmBH Schwäbisch, Germany), 1% polyethylene-block-poly (ethylene glycol, Sigma-Aldrich), and 1% SiO2 nanoparticle (10 nm in diameter, Meliorum Technology, Inc.) were precisely measured on a microbalance (Sartorius CPA225D) and mixed together in a 25 ml glass vial. PBP were manually grinded for 5 min for better dispersion in advance. One-percent nanoparticle concentration by weight has been used for consistency with the previous reports [19–26]. All the samples were sonicated (Branson 3510, Branson Ultrasonics Corporation) for 200 min to ensure homogeneous dispersion of nanoparticles and polar-ended organic molecule (PBP).

**Fig. 2:**
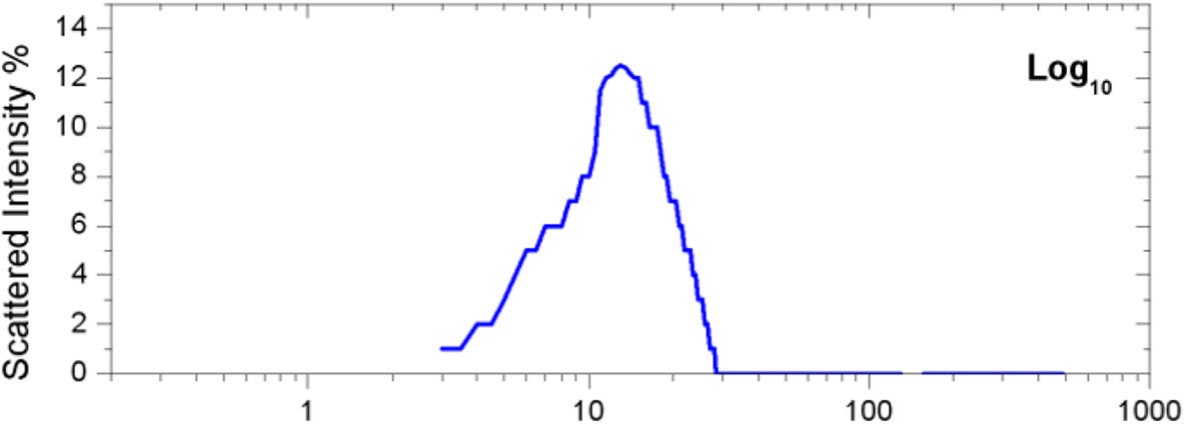
Nanoparticle size distribution by dynamic light scattering method

The actual nanoparticle size was measured by a photon correlation spectroscope and shown in Fig. 2. For 1000 mg of PAO superstructure, 980 mg of PAO-68, 10 mg of silica nanoparticle (1 wt.%), and 10 mg of PBP (1 wt.%) were prepared and measured on the microbalance. The same amount of PAO (1000 mg) was prepared to be used as a reference. PAO (990 mg) and silica nanoparticle (1 wt.%; 10 mg) mixture was also prepared to confirm any direct effect of silica nanoparticles on the effective specific heat of PAO; this control experiment may also be useful to confirm that enhanced specific heat by doping with nanoparticles can only be naturally possible in molten salts due to the unique nanostructure formation by salt molecules. In order to confirm the effect of PBP on PAO, we also prepared the same amount of PAO (990 mg) and 1 wt.% of PBP (10 mg). A modulated differential calorimeter (Q20, TA Instruments, Inc.) was employed to characterize the specific heat of each sample. Tzero Hermetic Pans/Lids were used to mount each sample to ensure no sample loss during the specific heat measurement. The weight of each sample was measured on a microbalance (CPA225D) before and after each measurement to ensure no mass loss. Each sample pan/lid was disposed after each experiment, in order to ensure no contamination between samples. For each test, the differential scanning calorimeter (DSC) curve was monitored to confirm no chemical reaction or no moisture effect during the specific heat measurement. For measuring the thermal conductivity, 4000 mg pure PAO and 4000 mg PAO nanofluid were prepared. The pure sample was tested first to get a reference value for the measurement. The chamber used for testing the thermal conductivity consists of two halves. The testing sample was poured into the lower half of the chamber. The upper half of the chamber was then placed over it. The flanges of the upper and lower half of the chamber were fixed with each other, and both halves of the chamber were fastened together with Allen screws. Eight thermocouples were inserted into the test chamber, and one was used to measure the furnace temperature. The thermocouples were connected to a data acquisition system (DAS) (National Instruments model: NI SCXI-1000). A hot wire was inserted into the center of the chamber. The ends of the hot wire were connected to a DC power source (Keysight Model: E3644A) and to the DAS. The test chamber was placed into an oven (Jeio Tech model: OV-11) set at 120 °C. The chamber was kept in the furnace for 2 h to reach steady state. After the wait, the DAS and the DC power supply were turned on. The power supply was set at 11 V DC, with current value of 2.5 A. The LabVIEW program was used to store the data. For measuring the viscosity, 2000 mg pure PAO and 2000 mg PAO nanofluid were prepared. We used a Discovery Hybrid Rheometer (HR-1) which has a cone-plate geometry. The cone-shaped bob is connected to the spindle drive and the sample will pour into the cup below the bob, and while rotating the bob, the different drag of the nanofluid is measured. This device was connected to the software Trios V.3.3.1.4246 in a computer to store data; it has its own calibration before starting any tests, and the protocol used is 1-h soaking time for each of two flow sweeps at 120 °C.

## Results and Discussion

### Specific Heat

**Fig. 3:**
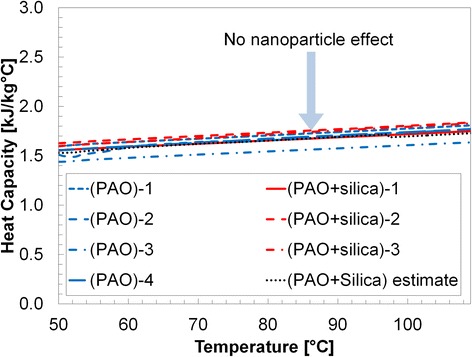
Specific heat measurement of pure PAOs and those with dispersed SiO_2_ nanoparticles (1 wt.%). The result shows no significant change in the specific heat. As reported in the literature [33, 34], the specific heat enhancement by nanoparticles seems only possible for molten salt nanofluids. The conventional effective specific heat model (Eq. 1) made a good agreement with the experimental data

**Fig. 4:**
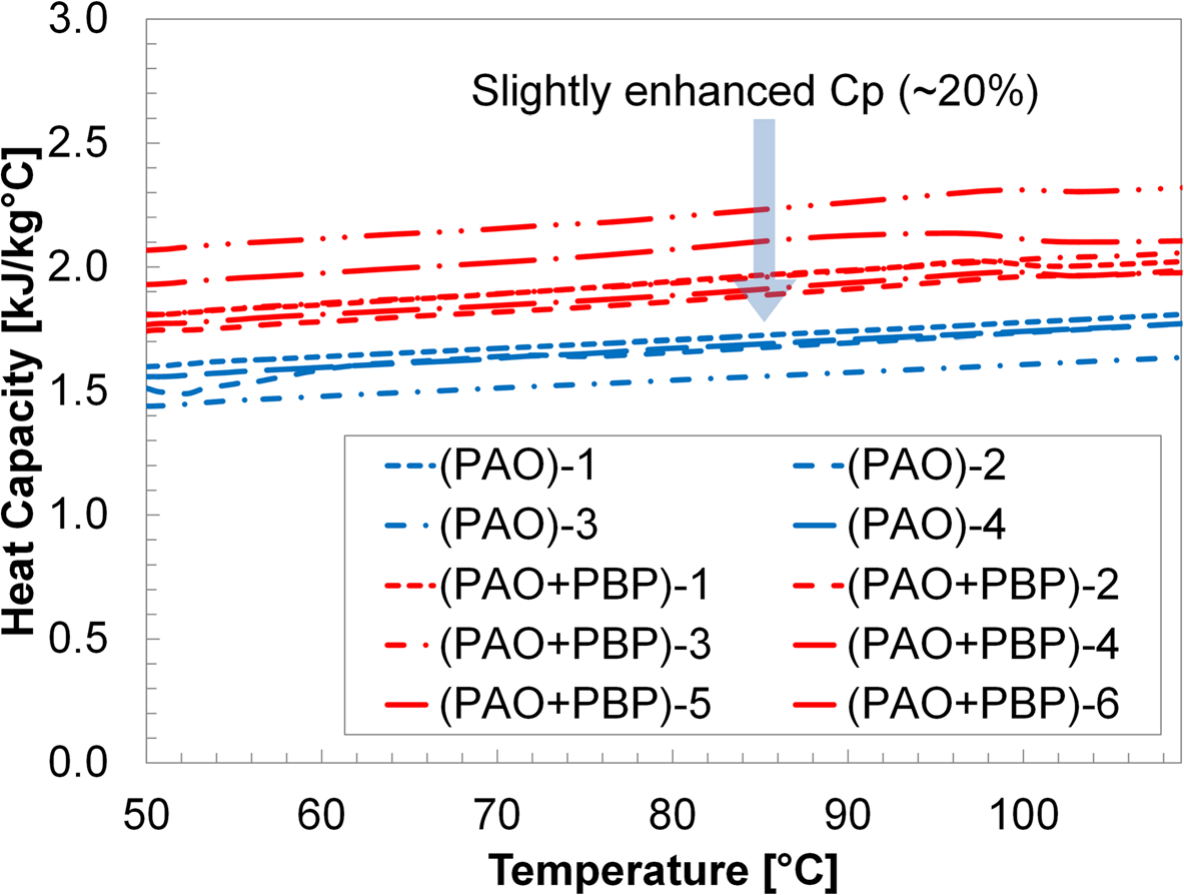
Specific heat measurement of pure PAOs and PAOs doped with proposed pre-fabricated nano-additives (i.e., polyethylene-block-poly (PBP) (1 wt.%)). Slightly enhanced specific heat was observed with a large variation in the measurement. The variation may result from a possible agglomeration of pre-fabricated PBP in the mixture

**Fig. 5:**
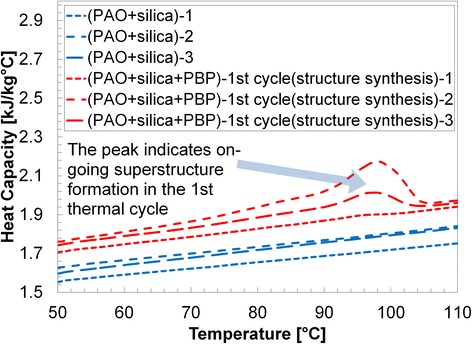
The initial DSC tests of the proposed pre-fabricated nano-additives (i.e., polyethylene-block-poly (PBP) (1 wt.%)) together with silica nanoparticles (1 wt.%) in PAOs. The dispersed PBPs are expected to ionically bond to the nanoparticles to form superstructure in the first thermal cycle. Small peaks observed near 100 °C may indicate the bonding process takes place in the thermal cycle (over 100 °C)

**Fig. 6:**
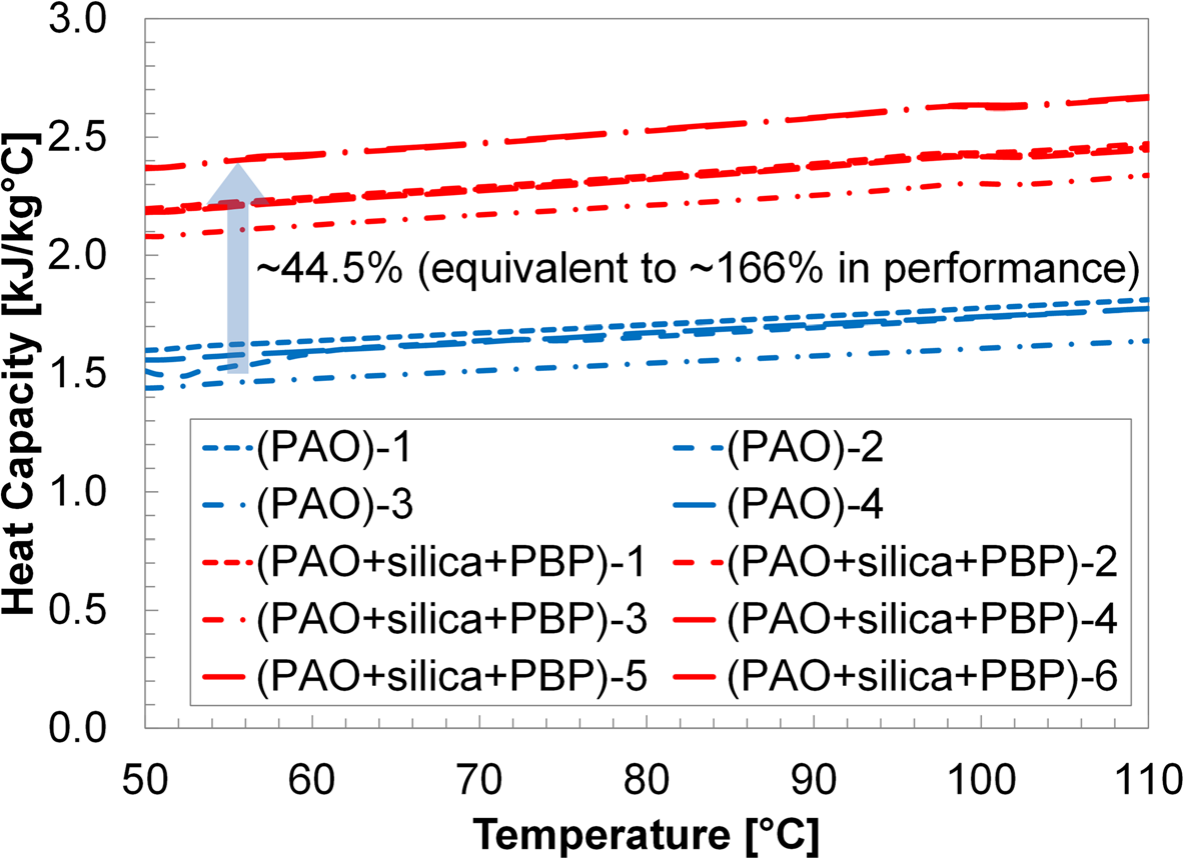
Specific heat measurements of PAO + SiO_2_ (1 wt.%) + PBP (1 wt.%) after the first thermal cycle. All the six samples have shown significant specific heat enhancement compared with the PAOs. The variation in the specific heat seems larger for the PAO superstructure than the pure PAO due to the presence of the nanoparticle and the nanostructure

**Fig. 7:**
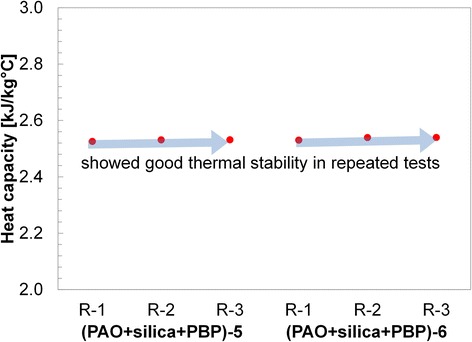
Specific heat measurement of PAO + SiO_2_ (1 wt.%) + PBP (1 wt.%) up to three repeats. No significant degradation of specific heat was observed. However, the stability of the superstructure with more cycles for a long-term stability test is needed in the future

Results are shown in Table 1 and Figs. 3, 4, 5, 6, and 7. Figure 3 shows the specific heat measurements of pure PAO and PAO + silica nanoparticle (1 wt.%) and the estimate by the conventional effective specific heat model (Eq. 1). Since the nanoparticle concentration is very low (~1% by weight), the estimate by the model [1] made a good agreement with the experimental data. It also supports the recent report [34] that specific heat enhancement doped with nanoparticles is not possible for PAO medium (i.e., non-salt based). Figure 4 shows pure PAO and fabricated nanostructure (PBP (i.e., hydroxy-ended poly (polyethylene-block-poly) (MW ca. 1400))) at 1% concentration by weight. Slightly enhanced specific heat (~20%) was observed with a large variation in the measurement. The variation may result from a possible agglomeration of pre-fabricated PBP in the mixture. Figure 5 shows PAOs and PAOs + silica nanoparticles (1 wt.%) + PBP (1 wt.%) in the first thermal cycle. All results showed a linear increase of specific heat with temperature. The dispersed PBPs are expected to ionically bond to the nanoparticles to form a superstructure in the first thermal cycle. Small peaks observed near 100 °C may indicate the bonding process takes place in the thermal cycle (over 100 °C). Figure 6 shows a specific heat of sample of PAO and PAO + silica nanoparticles (1 wt.%) + PBP (1 wt.%) after the first DSC thermal cycling test (hence, all PBPs can be completely bonded to nanoparticles). We observed the average of 44% enhanced heat capacity (2.37 kJ/kg °C) by using only 1% nanoparticles along with 1% PBP in comparison with pure PAO (1.65 kJ/kg °C) at 80 °C. A total of six samples have been separately synthesized and tested on different days. The standard error was only 0.05 kJ/kg °C. Moreover, the peak in Fig. 5 is vague in Fig. 6 which indicates that the in situ formation of the superstructure was completed in the first thermal cycle. Figure 7 shows a repeatability test. Samples #5 and #6 have been selected from Fig. 6 and repeated three times. The result shows a very good agreement between measurements, and no significant specific heat change was observed. The standard error was only 0.002 kJ/kg °C.

**Table 1:** Specific heat (c_p_) measurement at 80 °C

	PAO (Fig. 3)	PAO + SiO_2_ (Fig. 3)	PAO + PBP (Fig. 4)	PAO + SiO_2_ + PBP 1st cycle (Fig. 5)	PAO + SiO_2_ + PBP 2nd cycle (Fig. 6)
1st c_p_	1.71	1.66	1.93	1.83	2.33
2nd c_p_	1.66	1.73	1.87	1.94	2.34
3rd c_p_	1.54	1.72	1.95	1.89	2.21
4th c_p_	1.67	–	2.07	–	2.32
5th c_p_	–	–	1.89	–	2.52
6th c_p_	–	–	2.2	–	2.53
Average c_p_	1.64	1.70	1.99	1.88	2.37
Enhancement	–	3.7%	21.04%	14.6%	44.5%
Standard deviation	0.07	0.04	0.12	0.06	0.06

### Thermal Conductivity

**Fig. 8:**
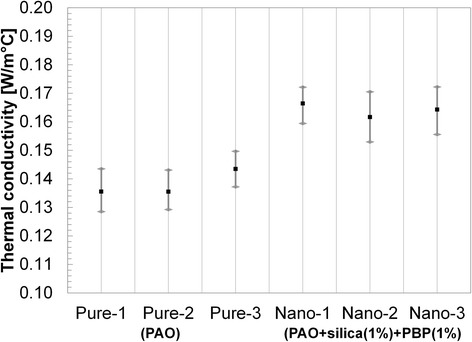
Thermal conductivity measurement of PAO and PAO superstructure (SiO_2_ (1 wt.%) + PBP (1 wt.%)). Around 20% enhanced thermal conductivity was observed

Pure PAO was tested three times to get a valid value, and the average value was found to be 0.138 W/m °C. Then the PAOs + silica nanoparticles (1 wt.%) + PBP (1 wt.%) were tested three times. The results are shown in Table 2 and Fig. 8. All the tests were performed at a constant temperature of 120 °C. The average *k* enhancement was measured to be ~19.8%.

**Table 2:** Thermal conductivity measurement at 120 °C

	PAO	PAO + SiO_2_ (1%) + PBP (1%)
1st test (W/m °C)	0.137	0.167
2nd test (W/m °C)	0.136	0.164
3rd test (W/m °C)	0.142	0.165
Average *k* (W/m °C)	0.138	0.165
Enhancement (%)	–	19.8
Standard deviation (W/m °C)	0.003	0.001

### Viscosity

**Fig. 9:**
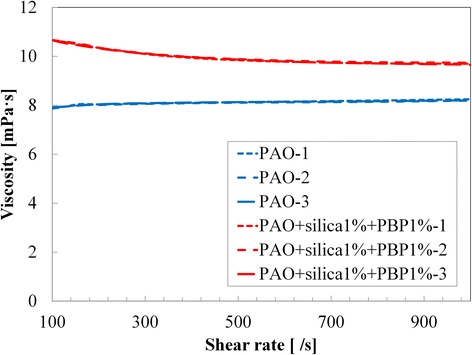
Viscosity measurement of PAO and PAO superstructure (SiO_2_ (1 wt.%) + PBP (1 wt.%)). Around 20% enhanced thermal conductivity was observed. The average viscosity increase is from 18% at the shear rate of 1000/s to 29% at the shear rate of 200/s. The shear thinning behavior is due to the presence of the superstructure, which has a high aspect ratio

First results were to obtain the pure PAO value. The temperature was set at 120 °C. Figure 9 and Table 3 show three repeats of pure PAO with a shear rate from 100/s to 1000/s. The viscosity of the pure PAO was observed to be independent of the shear rate and thus shows Newtonian behavior. The viscosity of PAO + SiO_2_ (1%) + PBP (1%) was observed to decrease with shear rate increase. This shear thinning behavior is due to the presence of nanoparticles together with PBP bonded to the nanoparticles. In comparison with pure PAO, the average viscosity increase is from 18% at the shear rate of 1000/s to 29% at the shear rate of 200/s (Table 3).

**Table 3:** Viscosity measurement at 120 °C

Shear rate (1/s)	PAO-1	PAO-2	PAO-3	PAO + silica (1%) + PBP (1%)-1	PAO + silica (1%) + PBP (1%)-2	PAO + silica (1%) + PBP (1%)-3
100	7.91	7.87	7.92	10.67	10.66	10.66
126	7.94	7.94	7.94	10.59	10.60	10.55
158	8.04	8.01	7.99	10.48	10.52	10.45
200	8.04	8.01	8.04	10.35	10.36	10.33
251	8.06	8.04	8.07	10.22	10.22	10.20
316	8.08	8.06	8.09	10.07	10.09	10.08
398	8.10	8.09	8.11	9.94	9.98	9.97
501	8.12	8.11	8.13	9.84	9.89	9.87
631	8.15	8.12	8.15	9.77	9.81	9.77
794	8.19	8.14	8.16	9.71	9.76	9.71
1000	8.24	8.19	8.20	9.67	9.71	9.63

### Figure of Merit

The enhancements in specific heat and thermal conductivity can improve the PAO’s heat storage and transfer performance. However, the viscosity increase may increase the pumping power, and thus, it is necessary to evaluate the effect of the proposed in situ synthesis of superstructure in PAO whether or not it is useful in thermal fluid applications. For heat storage performance, Bonilla [40] suggested a figure of merit to determine the performance of a given storage fluid by comparing the required pumping power to maintain the temperature difference between the inlet and the outlet of the storage fluid for forced convection in a turbulent regime, given by:

2 $$ {\mathrm{FOM}}_1=\frac{\rho^{2.0}{{\mathrm{C}}_{\mathrm{p}}}^{2.8}}{\upmu^{0.2}} $$

For heat transfer performance, Lenert [41] modified the Mouromtseff number to combine the effect on the conductive heat transfer coefficient in the radial direction and the effect of the thermal storage capacity in the axial direction in a turbulent flow which is as follows:

3 $$ {\mathrm{FOM}}_2=\frac{\rho^{2.0}{{\mathrm{C}}_{\mathrm{p}}}^{1.6}{K}^{1.8}}{\upmu^{1.4}} $$

Assuming the density is nearly constant, results of figure of merit (FOM) analyses are summarized in Table 4. Average specific enhancement of 44.5% and average thermal conductivity enhancement of 19.8% are substituted to Eqs. 2 and 3. Since the viscosity of the PAO superstructure shows a non-Newtonian behavior (i.e., shear thinning), the minimum increase of 18% at the shear rate of 1000/s and the maximum increase of 29% at the shear rate of 100/s are used to complete FOM_1_ and FOM_2_. Results show that the performance of PAO for heat storage can be enhanced by ~166–171% and for heat transfer by ~75–98%.

**Table 4:** Figure of merit analyses (FOM_1_ and FOM_2_)

Properties and figure of merit	Effect of superstructure (SiO_2_ (1%) + PBP (1%))
c_p_%	44.5%
*k%*	19.8%
*μ%*	29~18% (from 100/s to 1000/s)
FOM_1_ (for heat storage)	2.66–2.71
FOM_2_ (for heat transfer)	1.75–1.98

## Conclusions

In conclusion, recent studies [33, 34] proposed that doping oxide nanoparticles into a molten salt eutectic can induce salt molecules to form a fractal-like nanostructure on a nanoparticle and thus enhance the effective specific heat of the molten salt eutectic. This is due to a unique feature of molten salt eutectics since salt molecules are likely to electrostatically interact with oxide nanoparticles and settle down on their surfaces to form a nanostructure. Hence, such nanostructural formation can occur only in molten salt media, and thus, such specific heat enhancement of fluid is only naturally possible in molten salt media. In this study, we demonstrated that such nanostructures can be mimicked through in situ formation of fabricated superstructures, constructed by nanoparticles coated with pre-fabricated nanostructures (i.e., hydroxy-ended poly (polyethylene-block-poly) (MW ca. 1400), termed as “PBP”). We first applied this approach to polyalphaolefin (PAO), which is a well-known non-polar thermal fluid in aviation platforms and radar systems. A differential scanning calorimeter (DSC) was employed to characterize specific heat. Since these structure formations can also increase thermal conductivity and viscosity like nanofluids, these properties were also investigated. Results showed 44.5, 19.8, and 22.5% of specific heat, thermal conductivity, and viscosity enhancement, respectively, in comparison with pure PAO. In addition, the enhancements in specific heat and thermal conductivity can improve the PAO’s heat storage and transfer performance. However, the viscosity increase may increase the pumping power (negative effect), and thus, it is necessary to evaluate the effect of the proposed in situ synthesis of superstructure in PAO whether or not it is useful in thermal fluid applications. Hence, we evaluate its heat storage and transfer performance by using generalized figure of merit analyses. Results show that heat storage and transfer performances can be enhanced by ~166–177% and ~75–98%, respectively. This is the first demonstration of enhanced specific heat by in situ formation of nanostructure and may provide a new approach to organic molecule modification to the materials science community.

## Abbreviations

DSC: Differential scanning calorimeter
FOM: Figure of merit
PAO: Polyalphaolefin
PBP: Polyethylene-block-poly
